# Complete Exsection of the Median Nerve, with Restored Sensibility—Exsection of the Musculo-Spiral Nerve with Loss of Both Sensation and Motion—Division of the Ulner Nerve; Sensation and Motion Regained

**Published:** 1868-06

**Authors:** J. F. Miner


ART. III.—*Complete Exsection of the Median Nerve, with restored sensibility—Exsection of the Musculo-Spiral Nerve with loss of both sensation and motion—Division of the Ulner Nerve; sensation and motion regained.* BY J. F. MINER, M. D.

Some of our readers may have noticed in the *London Lancet* of last November, report of a case in the wards of Prof. Richet at *La Pitie*, of complete division of the median nerve, with preservation of sensibility, a case which is said to be exciting much interest and giving rise to considerable speculation on account of the numerons physiological problems that it involves. This case is now going the rounds of the various medical journals in this and other countries, and on this account I am led to report two or three cases of division and exsection of this nerve, and other large nervous trunks which have come under my care within the last year, believing them similar in some respects, and equally interesting in a physiological point of view.

The history of the case, as it is re-published in the *New York Medical Journal*, is briefly as follows: On the 23d of October, the patient, a female, aged 24, fell heavily on some sheets of copper, and was severely wounded at the wrist. She was taken to the Hôtel Dieu, where the radial artery was tied by the house surgeon. Twenty-four hours after M. Richet examined the wound. It measured six centimetres across the wrist, and was situated about six centimetres above the radio-carpal articulation. The superficial muscles were found divided. The radial artery was completely cut through, *as was also the median nerve*, and the flexor profundus itself bore marks of injury, yet sensibility of the lower end of the nerve was unimpaired. The patient screamed with pain as M. Richet excised a minute portion of it for microscopical examination. All the parts to which the median nerve is distributed retained their sensibility.

The following cases have come under my care, and two of them have been previously referred to by my friend and assistant, Dr.

C. F. A. Nichell, in the Buffalo Medical Association. The important questions involved had not so much attracted my attention until I observed how much speculation had been caused in Europe by the case in the wards at *La Pitie*, under Prof. Richet.

The first case which I desire to mention in this connection is Mr. W. C., who received accidentally a gun-shot wound in October, 1867, the ball passing directly through the median nerve about two inches above the condyles of the humerus. The wound healed, but the fingers became flexed, and five months after injury, false anchylosis had formed. The pain from receipt of injury was most intense and indescribable. At the end of the twenty-second week, when seen by me, the pain had become so unendurable that the poor man was begging to be shot, or in some way killed, in order to put an end to his sufferings; indeed he had made several efforts to help himself out of the world. The excruciating pain together with the large quantities of opium taken to relieve it, impaired digestion, producing great emaciation. An incision was made upon the inner margin of the biceps muscle, four or five inches in length, exposing the median nerve in its course over the brachial artery; the nerve was found closely involved in the cicatrix of the wound, and was with some difficulty dissected out; the surrounding tissues carefully separated and three inches of the nerve removed. The wound united rapidly, pain ceased immediately, and artificial motion was employed to restore the mobility of the joints of the hand and fingers which now are nearly as perfect as ever. *Sensation* has at no time been absent; there was at first some diminution of sensibility along the anterior portion of fore-arm and hand, but never complained of except in reply to careful questioning. Sensation and motion are now perfect.

It appears that some physiologists in Europe have suggested possible mistake, that the whole nerve might not have been divided in the case in *La Pitie.* In this case there can be no doubt. The specimen, now preserved, shows beyond all question that three inches of the median nerve has been removed. The question of union in nerve filliments when simply divided, is also excluded, and the only alternative is this: the median nerve does not so exclusively supply any of the integument or muscles as that when exsected, either sensation or motion is wholly destroyed.

*Exsection of the Musculo-Spiral Nerve.*—The second case proposed is that of Mr. M., who presented himself for removal of a tumor occupying the lower portion of the arm. The growth presented the general appearance of an encysted tumor, movable, elastic to the touch, and well defined. Its history extended over a period of five years, growing slowly, and at no time interfering with the functions of the arm, or causing any very great inconvenience until within the last two months, when it rapidly increased in size and became exceedingly tender on pressure, and finally too painful for endurance. An incision of three inches in extent over the tumor, and careful displacement of the tissues, revealed a cystic growth developed in and embraced or surrounded by the nerve fibres. Finding it impossible to separate the tumor from the nerve, the nerve was divided both above and below the tumor, when it was easily separated from the tissues and removed. The extensor muscles of the hand and fingers were paralyzed, and numbness was felt over the whole back portion of the fore-arm and hand. Exsection of the musculo-spiral nerve, one of the largest nerve trunks in the arm, had been made, and was followed by loss of motion and in great degree of sensation in all the parts it supplies.

*Division of the Ulner Nerve.*—Mr. M. N., in March, 1868, fell upon some glass-ware and divided all the flexor tendons of the middle, ring, and little finger, the ulner artery and *nerve.* The artery was ligated on both sides of the division; the wound approximated with sutures and united rapidly. The tendons were involved in the cicatrix and did not allow motion in the tendons except so far as permitted by the movement up and down of the whole cicatrix. Sensation was nearly lost immediately after reciept of injury, but has been perfectly regained, showing union of the nerve.

It would appear from these facts that the *median nerve* does not exclusively supply any portion of the hand or arm so exclusively as to make its removal inconsistant with almost perfect sensation and motion. In the above instance it was followed by impaired but not destroyed sensation, while motion remained normal. The *musculo-spiral nerve* was exsected, followed by greatly impaired sensation and loss of motion in the parts supplied. Division of the ulner nerve impaired sensation, almost destroyed it, but that motion is lost is not shown, since the tendons were involved in the cicatrix and flexion of the fingers would not have remained even if the nerve had not have been divided.

It is no part of the intention in reporting the above cases to draw any conclusions as to the functions of nerves, their re-union after division, or the capacity of nature to afford nervous supply from neighboring trunks when the original one has been in any way interrupted or destroyed; to relate the main facts, as briefly and accurately as possible, comprises the whole purpose. The practical and physiological questions which may be involved will be left for others, or for future consideration.

